# Growing inequities in maternal health in South Africa: a comparison of serial national household surveys

**DOI:** 10.1186/s12884-016-1048-z

**Published:** 2016-09-01

**Authors:** Njeri Wabiri, Matthew Chersich, Olive Shisana, Duane Blaauw, Helen Rees, Ntabozuko Dwane

**Affiliations:** 1 Epidemiology and Strategic Information Unit, Human Sciences Research Council, Pretoria, South Africa; 2 Wits Reproductive Health and HIV Institute, Faculty of Health Sciences, University of the Witwatersrand, Johannesburg, South Africa; 3 Evidence Based Solutions, Cape Town, South Africa; 4 Department of Psychiatry and Mental Health, University of Cape Town, Cape Town, South Africa; 5 Centre for Health Policy, School of Public Health, Faculty of Health Sciences, University of the Witwatersrand, Johannesburg, South Africa

**Keywords:** Maternal health, South Africa, Equity gap, Access, National household survey, Relative inequalities

## Abstract

**Background:**

Rates of maternal mortality and morbidity vary markedly, both between and within countries. Documenting these variations, in a very unequal society like South Africa, provides useful information to direct initiatives to improve services. The study describes inequalities over time in access to maternal health services in South Africa, and identifies differences in maternal health outcomes between population groups and across geographical areas.

**Methods:**

Data were analysed from serial population-level household surveys that applied multistage-stratified sampling. Access to maternal health services and health outcomes in 2008 (*n* = 1121) were compared with those in 2012 (*n* = 1648). Differences between socio-economic quartiles were quantified using the relative (RII) and slope (SII) index of inequality, based on survey weights.

**Results:**

High levels of inequalities were noted in most measures of service access in both 2008 and 2012. Inequalities between socio-economic quartiles worsened over time in antenatal clinic attendance, with overall coverage falling from 97.0 to 90.2 %. Nationally, skilled birth attendance remained about 95 %, with persistent high inequalities (SII = 0.11, RII = 1.12 in 2012). In 2012, having a doctor present at childbirth was higher than in 2008 (34.4 % versus 27.8 %), but inequalities worsened. Countrywide, levels of planned pregnancy declined from 44.6 % in 2008 to 34.7 % in 2012. The RII and SII rose over this period and in 2012, only 22.4 % of the poorest quartile had a planned pregnancy. HIV testing increased substantially by 2012, though remains low in groups with a high HIV prevalence, such as women in rural formal areas, and from Gauteng and Mpumalanga provinces. Marked deficiencies in service access were noted in the Eastern Cape ad North West provinces.

**Conclusions:**

Though some population-level improvements occurred in access to services, inequalities generally worsened. Low levels of planned pregnancy, antenatal clinic access and having a doctor present at childbirth among poor women are of most concern. Policy makers should carefully balance efforts to increase service access nationally, against the need for programs targeting underserved populations.

**Electronic supplementary material:**

The online version of this article (doi:10.1186/s12884-016-1048-z) contains supplementary material, which is available to authorized users.

## Background

South Africa is one of the most inequitable countries in the world, by almost any measure. The wealthiest 10 percent of the population, for example, accounts for more than half the country’s income [1]. Indices of health, and especially of maternal health, clearly reflect the inequalities in access and health outcomes that mark the country. Maternal mortality varies considerably between provinces, for instance, with the institutional-level maternal mortality rates (MMR; maternal deaths per 100,000 live births) ranging from 69 in the Western Cape to 185 in the North West Province [2]. The prevalence of HIV also differs substantially between geographical areas. The 2012 national antenatal survey found that the HIV prevalence at district level ranges from 1.5 to 40.7 %, around a national average of 29.2 %[3].

Maternal mortality has fluctuated over the past decades. Institutional MMR progressively escalated from the late 1990s onwards, up to levels of 176 nationally in the 2008–2010 triennium, but dropped thereafter to 147 by 2012 [4, 5]. The wide scale-up of antiretroviral treatment (ART) across the country, and especially among pregnant women, is credited with these reductions [5, 6]. While HIV-related mortality has decreased, maternal deaths due to haemorrhage have actually risen, especially among women who had a caesarean section [5]. The increase in these and other deaths from direct obstetric causes, are ascribed to deficiencies in the quality of maternal health services, most notably in patient transport, the availability of intensive care units and the provision of emergency obstetric care [5].

Health service data are available to guide improvements in the quality of services. The district monitoring systems have been strengthened [2] and the factors contributing to maternal deaths are frequently assessed [4, 6, 7]. What we lack, however, is an assessment of access to maternal health services at a population level, disaggregated by population group, and assessed over time. Analysing data from national household surveys can fill these gaps. A survey in 2008 [8] highlighted marked differences in maternal health status across socio-economic and other population groups, especially between rural and urban areas. Using data from a follow-up survey in 2012, the study presented here updates the 2008 findings and examines how inequalities have changed since then. The analysis focuses primarily on absolute and relative differentials between socio-economic groups, but also on the influence of factors such as rural–urban location, race and HIV status. More broadly, by determining the distribution and outcomes of maternal health services over time, the study identifies the underserved groups and geographical areas. This information can be used to help direct health system resources and initiatives to raise the quality of services.

## Methods

### Survey sampling, field and laboratory procedures

This paper is a sub-analysis of the third (2008) and fourth (2012) South African National HIV Prevalence, Incidence, Behaviour and Communication Surveys [9, 10]. The two surveys employed multistage stratified sampling, taking into account province; locality (urban formal, urban informal, rural formal including commercial farms, and rural informal or tribal areas); and race groups. Full details of the survey methods, response rates and ethical procedures are detailed elsewhere [9, 11]. In brief, the sampling frames were based on enumerator areas (EAs) used in the South Africa national census. The primary sampling units consisted of 1000 EAs, which were selected from a database of 86,000 EAs. Fifteen households within each selected EA constituted the secondary sampling units (15,000 households). The same EAs were used in both surveys, but different households were selected. The final sampling unit was made up of eligible individuals within households. Anyone who slept in the household on the night preceding the survey (including visitors) was considered a household member. In 2008, only four persons were eligible to participate from each household; one in each age group (0–1, 2–11, 12–14, and above 15 years) [8]. In the 2012 survey, all persons in the selected households were eligible. Consenting participants responded to individual questionnaires. Dried blood spot specimens were collected from consenting participants, tested for HIV antibodies and linked anonymously with the questionnaires administered to study participants [12].

### Study variables and measures

The analysis includes data collected from two groups of women aged 15–55 years: those who had been pregnant in the preceding 2 years and those interviewed as the parent or guardian of a child below two years. The study variables are described in the publication of the 2008 survey findings [8], and are only overviewed here. Socio-economic quartiles (SEQ) were derived from an asset score based on measures of household-living standards, and were generated using multiple correspondence analysis [13, 14]. Quartiles were preferred over quintiles as the socio-economic differentials are very narrow in many areas of the country, given that many women perform the same income-generation activities and thus have similar incomes and asset levels [8, 15]. Quintiles would thus have been unable to differentiate between women in Q1 and Q2, who have essentially the same living standards.

Access to maternal health services was measured by: utilisation of antenatal clinics; HIV testing coverage; and the presence of a skilled birth attendant (SBA) or doctor at birth [8]. Maternal health status was not assessed in detail within the survey, thus proxy indicators were used. Women who said they had a fair or poor health status were categorised as having a lower self-assessed health status, and compared with those reporting good or excellent health. Planned pregnancy, multiparity (five or more children), and prevalence of HIV infection were used as indicators of maternal health status, given their links with pregnancy outcomes for women and children [16].

Though the study focused on the distribution of access and outcomes by SEQ, variation was also assessed across other categories of social differentiation. The applicable categories of the PROGRESS-Plus acronym were used, namely: **P**lace of Residence (province; locality as urban formal and informal, and rural formal and informal), **R**ace, **O**ccupation, **E**ducation, **S**ocio-economic Status (SEQ and employment of the mother), and Age and HIV status representing the **Plus** category [17]. We also examined whether there were systematic differences in access to services between those with and without HIV infection.

### Statistical models and measures of inequality by socioeconomic status

Data were analysed using Stata version 13.0 (College Station, Texas, United States [18]), taking into account the complex multilevel sampling design (by age, race group and province) and participant non-response. Summary indices for descriptive analysis are weighted percentages, while unweighted counts are provided. Clustering was not accounted for given that the large number of primary sampling units (1000) in the study is comparable to respondent number, diminishing such effects [8].

Socio-economic inequalities in maternal health were calculated using three inequality measures: the Slope Index of Inequality (SII) for quantifying absolute inequalities, and the Relative Index of Inequality (RII) and Concentration Index (CI) for assessing the magnitude of relative inequalities [19–21]. ArcGIS Desktop Version 10.0 was used to show the geographical variation in access to antenatal services and a skilled birth attendant, planned pregnancy and health status.

### Slope index of inequality and the relative index of inequality

The SII and RII indices are regression based and take the whole wealth distribution into account, rather than only comparing the two most extreme groups (e.g., the wealthiest and poorest quartiles), such as done with a rate difference and rate ratio [20]. The RII and SII are “recommended when making comparisons over time or across populations” [22, 23]. While most trend studies focus on relative, as opposed to absolute inequalities [24], the use of both provides a more complete assessment of patterns of inequalities and changes over time [25, 26].

To derive the SII and RII, each woman in the study population was assigned a notional socio-economic rank score, scaled to take values between 0 (bottom of hierarchy (Q1)) and 1 (top of hierarchy (Q4)) [8]. The rank score equals the midpoint of the range in the cumulative distribution of the population of participants in a given SEQ [24]. For example, if the Q1 women comprise 34.5 % of the population, the women in this category are assigned a rank score of 0.17 (0.345/2), and if the Q2 women comprises 32.5 % of the population, the corresponding rank score is 0.51 (0.345 + [0.325/2]) and so forth. The generated individual data is self-weighted and the only weight applied in the analysis is the survey weight to correct for survey sample design.

We then used generalised linear models (GLM) to fit binomial models (Eq. 1) to generate inequality measures, as has been suggested by several authors [24, 27–30].

1 $$ g(Y)={\beta}_0+{\beta}_1 rscore+{\beta}_2 survey+\varepsilon $$

where Y = 1 if outcome is present and Y = 0 if absent, *g(Y) = Y* is the identity link function (i.e. binomial regression) generating SII together with the 95 % confidence and, *g(Y) = log(Y)* is log link function (i.e. log-binomial regression) generating the RII and the 95 % confidence interval, *rscore* is the notional socio-economic rank score for each woman, *β*_1_ and *β*_2_ are the regression coefficients. *Survey* equals 1 for the 2008 survey and 2 for the 2012 survey, and *ε* is the error term with a binomial distribution.

The SII, the *β*_1_ under binomial regression, represent the estimated difference in predicted value of the outcome between those at the top (wealthiest) and those at the bottom (poorest) of the social hierarchy. SII is the absolute effect on health outcome of moving from the poorest to the wealthiest group [20, 31]. A positive SII represent inequality in favour of the wealthy, while a negative SII is inequality in favour of poor.

The RII, the exponential of the slope, exp(*β*_1_) under log-binomial regression, represents the proportionate difference in outcome across the distribution of socio-economic position; or the likelihood of having an outcome, relative to one’s SES level. The RII increases from zero, with higher values indicating higher inequality. An RII above one indicates that the outcome is more prevalent among wealthy women, compared to their poor counterparts. To deal with lack of model convergence, we fitted Poisson regression with robust variance [32]. Poisson regression is suitable when outcomes are not rare, as in this study where most had prevalence greater than 10 %[28]. A decline in both SII and RII is the best evidence of progress in closing the inequality gap [24].

For each outcome, the linear trends of the RII and SII over the five year period 2008–2012 were tested by estimating the p-value for an interaction term between rank score and years since baseline, i.e. 2008 survey coded 1, 2012 coded 2, to account for the different time intervals between surveys (Eq. 2). Positive and significant coefficients, *β*_3_ greater than one, for the interaction term indicate widening RII (SII) inequalities over time.

2 $$ g(Y)={\beta}_0+{\beta}_1 rscore+{\beta}_2 survey+{\beta}_3 rscore* survey+\varepsilon $$

### Concentration index

The concentration index is defined with reference to the concentration curve, which plots the cumulative proportion of health outcome against the cumulative proportion of the population, ranked by SEQ beginning with the poorest [8, 33]. If health access is equally distributed across SEQ, concentration curves coincide with the diagonal line of equality. The Concentration Index is given by twice the area between the concentration curve and line of equality and ranges from −1 to 1. Zero represents perfect equality, while positive values indicate richer individuals have greater coverage (or good health outcomes) than poorer individuals [33].

## Results

In 2008, data were gathered on 23,308 people, and 56.0 % of those interviewed were women aged 15–55 years (8292/14,798). Of these women, 13.5 % (1121/8292) had been pregnant in the preceding 2 years, and 15.8 % (1310/8292) had a child in the past 2 years. The proportion of survey participants aged 15–55 years in 2012 was similar to that in 2008 (53.5 %; 13,187/24,659). Of these, 12.5 % (1648/13,187) had been pregnant or had a child in the past 2 years.

### Variation in socio-economic characteristics of women by population group

In both surveys, about 95 % of pregnancies occurred in African or Coloured women (Table 1). White and Indian women, however, constituted 33.6 % of the wealthiest quartile in 2012, higher than levels in 2008 (23.9 %). In each survey, about 60 % of women in QI and QII were single, while this figure was only 40 % among the wealthiest women. Wealth quartile remains strongly associated with educational attainment and employment. Overall, educational attainment appears to be rising (44.2 % of women had completed secondary school in 2012, versus 40.0 % in 2008). However, still only about 10 % of the population had any post-school education in 2012. The proportion unemployed and seeking work was higher in 2012 than in 2008 in QI (51.6 versus 44.0 %) and QII (49.2 versus 33.9 %), while it decreased in the two wealthier quartiles.

**Table 1:** Distribution of socio-economic status among women pregnant in past 2 years between 2008 and 2012; Distribution of socio-economic status among population sub-groups in women pregnant in past 2 years: analysis of the 2008 and 2012 national SABSSM survey

	Socio-economic Status								Overall total (Unweighted *N*, %)	
	QI poorest (%)		QII (%)		QIII (%)		QIV wealthiest (%)			
	2008	2012	2008	2012	2008	2012	2008	2012	2008	2012
Age categories									1111	1619**
15 to 19	12.1	14.2	13.1	9.5	9.6	9.9	4.6	4.6	11.1	10.2
20 to 29	50.4	50.8	48.6	57.8	52.4	55.3	51.4	50.6	50.5	54.0
30 to 39	32.8	26.2	36.2	28.9	35.5	31.4	37.0	39.8	35.0	30.4
40 to 55	4.7	8.8	2.1	3.8	2.4	3.5	6.9	4.9	3.4	5.3
Place of residence									1115***	1619***
Urban formal	7.8	11.3	27.6	27.1	82.0	76.8	95.7	89.7	43.2	45.7
Urban informal	20.3	11.0	16.3	12.0	7.6	9.1	1.5	1.0	13.7	9.3
Rural formal	10.9	4.8	9.7	3.1	6.3	2.9	0.3	6	8.3	3.9
Rural informal	61.0	72.8	46.4	57.8	4.2	11.3	2.5	3.3	34.8	41.1
Province									1115***	1619***
Eastern Cape	21.2	30.9	8.2	7.1	8.5	4.6	15.0	2.3	12.8	12.6
Free State	3.5	3.2	6.6	5.8	4.7	6.4	3.2	6.9	4.8	5.4
Gauteng	10.5	8.5	8.2	18.3	41.2	40.6	40.4	50.5	21.3	26.4
KwaZulu Natal	23.2	20.9	28.7	26.2	16	12.3	9.9	10.0	21.7	18.4
Limpopo	22.4	19.5	15.0	15.4	2.8	3.7	2.9	2.5	12.7	11.4
Mpumalanga	8.0	6.5	10.4	12.9	3.4	7.0	8.5	3.7	7.5	8.1
North West	7.8	6.8	13.7	8.1	8.2	6.0	1.3	4.7	9.2	6.6
Northern Cape	1.2	1.2	1.7	1.2	2.3	5.1	2.1	3.9	1.8	2.7
Western Cape	2.1	2.6	7.4	4.9	12.9	14.3	16.7	15.5	8.2	8.4
Race									1110***	1615***
African	98.4	96.9	95	95.7	77.5	82.9	60.4	49.7	88	85.7
White	0.0	0.1	0.3	0.0	8.1	2.0	17.6	23.4	4.0	4.0
Coloured	1.6	3.0	4.5	4.2	13.0	13.2	15.7	16.7	7.1	8.2
Indian	0.0	0.0	0.1	0.1	1.4	1.8	6.3	10.2	1.0	2.0
Highest education									1111***	1471***
No schooling-Gr3	7.1	3.6	5.3	2.5	1.7	1.2	0.2	1.2	4.4	2.3
Gr4-Gr7	17.8	19.0	10.1	7.9	5.4	4.2	2.8	1.8	10.4	9.2
Gr8-Gr11	58.3	60.9	51.4	44.0	34.4	40.0	13.8	20.5	45.3	44.4
Gr12	15.2	16.4	28.6	39.9	44.0	41.6	36.2	44.1	29.7	34.2
Tertiary	1.7	0.1	4.6	5.7	14.6	13.0	47.0	32.4	10.3	10.0
Employment									1096***	1589***
Housewife	26.2	17.0	21.1	11.2	13.8	12.5	11	17.5	19.6	14.2
Unemployed, not seeking work	9.6	10.9	10.0	7.6	3.4	7.0	2.0	2.3	7.3	7.6
Unemployed, seeking work	44.0	51.6	33.9	49.2	36.2	35.6	28.1	12.7	37.2	40.7
Informal sector, self-employed	4.6	0.6	6.0	3.4	6.3	7.2	9.6	7.0	6.0	4.2
Scholar or student	5.6	11.9	13.3	10.1	5.6	10.1	2.4	4.9	7.7	9.8
Part employed	6.5	3.6	4.1	5.5	4.0	7.7	4.0	14.2	4.8	6.8
Full employed	2.4	2.5	10.7	11.1	28.9	18.3	43.0	38.4	16.2	14.7
Pensioner, sick or disabled	1.2	1.9	1.0	1.9	1.8	1.7	0.1	3.0	1.2	2.0
Marital status									1113**	1601***
Single	59.3	57.8	64.8	62	55.7	50.2	41.1	36.1	58.4	53.7
Married/cohabiting	36.5	38.7	32.7	35.6	43.2	48.2	55.0	63.5	38.9	44.1
Divorced/widow	4.1	3.5	2.5	2.4	1.2	1.6	3.9	0.5	2.7	2.2

Distribution of socio-economic status among population sub-groups in women pregnant in past 2 years: analysis of the 2008 and 2012 national SABSSM survey. Table shows column percentages. *P* value assesses the distribution of population group across quartiles; **P < 0.01; ***P < 0.001

Socio-economic status varies considerably between provinces, and across urban and rural divides. In each survey, pregnant women in the wealthiest two quartiles were heavily concentrated in the urban formal areas of Gauteng and Western Cape. By contrast, almost three quarters of the poorest quartile lived in rural informal locations in 2012, up from 61.0 % in 2008. In the 2012 survey, the poorest quartile mostly lives in KwaZulu Natal (20.9 %), Eastern Cape (30.9 %) and Limpopo (19.5 %). Few wealthy women inhabit the latter two provinces (they together constituted only about 5 % of the wealthiest quartile in 2012).

### Inequalities in access to maternal health services

**Fig. 1:**
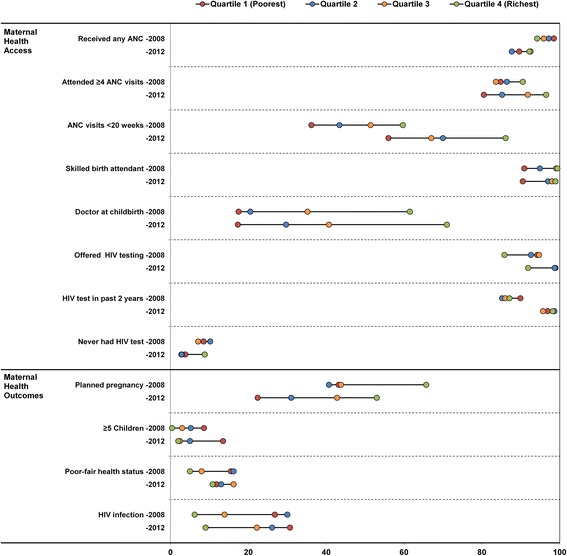
Time differentials in coverage of maternal health services and health status in South Africa; Time differentials in coverage of maternal health services and in maternal health status across socio- economic quartiles in South Africa

About 10 % of the overall population had not accessed ANC in 2012. Compared with 2008, *receipt of any ANC* declined in 2012 among all women and across almost all sub-groups, but especially in QI and QII women (Additional file 1: Table S1 and Fig. 1). Among those who attended ANC, the overall proportion that had *four or more ANC visits* was similar in 2008 and 2012, but inequalities within many population groups worsened. The gap between the poorest and wealthiest quartile rose from 5.7 % in 2008 to 16.0 % in 2012. In 2012, the proportion attending four or more visits rose with age and with each increase in education level (for example, from 72.9 % in those with under three years of schooling to 94.7 % in those with tertiary education), and was much higher in employed than unemployed women.

In 2012, considerably more women attended *ANC before 20 weeks* than in 2008, with double digit increases seen in most population groups, even in the poorest quartile. Though no changes were detected in inequalities over time, the SII (0.31) and RII (1.59) remained very high in 2012. Differences in 2012 were above 20 % between the sub-categories of almost all of the PROGRESS-Plus groups. Moreover, significant differences between more and less vulnerable groups were detected in early attendance for nine population sub-groups in 2012, but for only three in 2008 (Additional file 1: Table S1). In the 2012 survey, ANC attendance before 20 weeks was particularly low among women under 20 years, those living in rural and urban informal areas, and in Black African women. Importantly, 75.2 % of HIV-infected women attended before 20 weeks, compared to 63.5 % of uninfected women.

The substantial levels of absolute and relative inequality in *SBA coverage* were maintained in the second survey. Relatively low SBA coverage persists in QI, Black African women, rural areas and low education groups. Based on all measures of socio-economic inequality, *doctor attending childbirth* remains the most unequal of measures (CI = 0.27 in 2012, for example). Overall, the proportion of deliveries attended by a doctor had, however, increased considerably in 2012 in almost population groups, but was accompanied by a worsening of absolute inequalities (QI-QIV range and SII). The SII measure shows a 58.7 percentage point increase between the lowest and highest income quartiles. Gaps between race groups also rose (90 % of White and Indian women had a doctor present in 2012, 20–30 % higher than in 2008, while in Black African women, these levels only increased from 23.5 % in 2008 to 28.4 % in 2012).

Marked increments were noted in *HIV testing* in 2012, with these services largely pro-poor. More than 90 % of all population sub-groups had an HIV test in the past two years, considerably higher than in 2008. Of concern, however, testing levels were relatively low among some groups that had the highest HIV prevalence. For example, the following groups had an HIV prevalence above 20 % and more than 5 % had never tested: women 40–55 years, housewives, married or cohabiting women, unemployed women not seeking work, those who did not complete primary school, and women living in rural formal areas, or Gauteng and Mpumalanga Provinces. Still in 2012, some 3.9 % of HIV-infected women said they had never tested.

### Distribution of planned pregnancy and fertility

The overall proportion of *pregnancies that were planned* declined from 44.6 % in 2008 to 34.7 % in 2012, with the largest drop amongst the poorest quartile (Additional file 2: Table S2 and Fig. 1). Further, in 2012, more considerable step-wise decreases were noted in the proportion of planned pregnancy with each SEQ, and the rises in SII and RII indicate growing inequalities in this outcome (RII = 1.4 in 2008 and 3.0 in 2012; and SII = 0.13 in 2008 and 0.39 in 2012; Table 2).

**Table 2:** Trends in SII and RII in access to maternal health services and in health status; Trends in absolute and relative socio-economic inequalities in access to maternal health services and in maternal health status in South Africa

Variable	Range (Q4–Q1)		Slope of index inequality (95 % CI)			Relative index of inequality (95 % CI)			Concentration Index	
	2008	2012	2008	2012	Change in equity (*P*-value)	2008	2012	Change in equity (*P*-value)	2008	2012
Received any ANC	–4.3	2.6	−0.047** (−0.094 - -0.001)	0.051 (−0.027 - 0.130)	0.030	0.951** (0.904 - 1.000)	1.056 (0.971 - 1.149)	0.037	−0.005	0.015
Attended ≥4 ANC visits	5.7	16.0	0.024 (−0.084 - 0.132)	0.209*** (0.129 - 0.288)	0.005	1.027 (0.909 - 1.160)	1.271*** (1.139 - 1.417)	0.008	0.011	0.033
ANC visit <20 weeks gestation	23.5	30.1	0.274*** (0.106 - 0.442)	0.312*** (0.165 - 0.460)	0.741	1.831*** (1.268 - 2.644)	1.593*** (1.278 - 1.984)	0.525	0.116	0.077
Skilled birth attendant	8.5	8.4	0.121*** (0.066 - 0.176)	0.113** (0.020 - 0.207)	0.888	1.135*** (1.071 - 1.202)	1.124** (1.021 - 1.238)	0.869	0.017	0.018
Doctor attended childbirth	44	53.7	0.401*** (0.270 - 0.531)	0.587*** (0.449 - 0.724)	0.063	5.285*** (2.971 - 9.398)	5.802*** (3.856 - 8.728)	0.797	0.300	0.268
Offered HIV testing in pregnancy	−8.4	−7.0	−0.042 (−0.106 - 0.021)	−0.042 (−0.093 - 0.009)	0.999	0.951 (0.880 - 1.028)	0.940 (0.862 - 1.024)	0.833	−0.007	−0.004
HIV test in past 2 years	–2.8	1.3	−0.050 (−0.152 - 0.051)	−0.004 (−0.049 - 0.041)	0.419	0.943 (0.838 - 1.061)	0.996 (0.951 - 1.042)	0.405	0.000	−0.006
Never had HIV Test	–1.4	5.0	−0.028 (−0.120 - 0.065)	0.030 (−0.031 - 0.091)	0.297	0.739 (0.264 - 2.068)	2.631 (0.395 - 17.518)	0.236	−0.001	0.450
Planned pregnancy	22.5	30.6	0.134 (−0.032 - 0.301)	0.391*** (0.258 - 0.524)	0.009	1.391 (0.929 - 2.084)	2.999*** (2.096 - 4.292)	0.004	0.078	0.161
Five or more children	–8.2	−11.5	−0.099*** (−0.139 - -0.060)	−0.117*** (−0.162 - -0.073)	0.548	0.129*** (0.033 - 0.497)	0.045*** (0.014 - 0.144)	0.255	−0.326	−0.324
Poor-fair health status	–10.5	−1.0	−0.150*** (−0.234 - -0.066)	0.022 (−0.068 - 0.112)	0.004	0.331*** (0.167 - 0.654)	1.165 (0.631 - 2.151)	0.004	−0.043	−0.040
HIV infection	–20.6	−21.7	−0.281*** (−0.427 - -0.136)	−0.251*** (−0.372 - -0.129)	0.38	0.340*** (0.178 - 0.649)	0.372*** (0.224 - 0.620)	0.821	−0.193	−0.213

Trends in absolute and relative socio-economic inequalities in access to maternal health services and in maternal health status in South Africa: the repeated cross-sectional national SABSSM surveys 2008 and 2012

** *P* < 0.01; ***P < 0.001

In both surveys, only about a third of HIV-infected women reported having had a planned pregnancy. In 2012, levels of unplanned pregnancy were highest in informal areas (both rural and urban ones), which have the highest HIV prevalence of all places of residence. Among women younger than 20 years in the second survey, only 8.7 % of pregnancies were planned. Scholars and students accounted for 9.8 % of all pregnancies in 2012, while only 7.7 % in 2008.

The markedly unequal distribution of *multiparous women* across socio-economic groups persists: in 2012, 13.5 % of QI women had a parity of five or more, while this figure was 6.4 % in QIV women (Fig. 1). Also, only 3.5 % of women in formal urban areas had five or more children, compared with about 9.0 % in rural areas.

### Inequalities in maternal health status and in prevalence of HIV infection

Overall, in both surveys, around 13 % of women described themselves as being in *poor or fair health*. However, unlike in 2008, the proportion of women reporting this outcome in 2012 was very similar between SEQs, thus reflecting a narrowing of relative and absolute inequalities over time (Table 2). The proportion of HIV-infected women with a poor-fair health status was lower in 2012 (20.2 %) than in 2008 (25.1 %; Additional file 2: Table S2). In 2012, however, the proportions with poor or fair health increase stepwise with age category or decrease in education group.

The distribution of HIV infection among population groups in 2012 remained markedly unequal, though inequalities between SEQs were stable. HIV status is strongly associated with demographic and socio-economic characteristics – in both surveys, there are double digit absolute percentage differentials within each of the PROGRESS-Plus groups.

### Inequalities in access and outcomes between different provinces

**Fig. 2:**
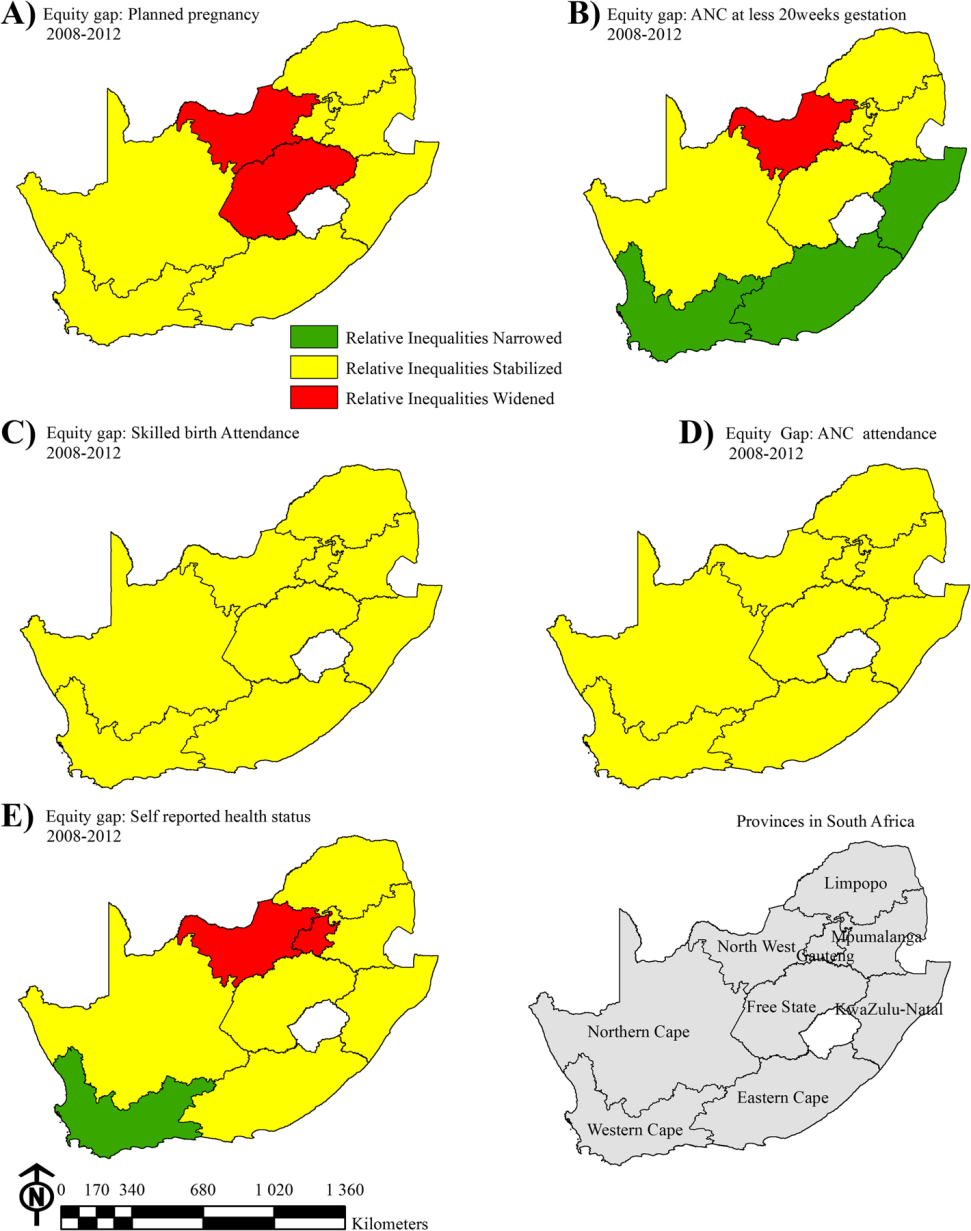
RII differentials in Planned pregnancy, SBA, ANC, health status, ANC at less 20 weeks gestation; Provincial differentials in RII in Planned pregnancy, SBA, ANC, health status and ANC at less than 20 weeks gestation over 5 years period in South Africa. Yellow represent provinces with stable high RII, Green indicates provinces where RII narrowed, an improvement, and red indicate provinces where RII widened indicating a high equity gap in access between wealthy and poor women

Large differences were noted at provincial level for almost all the indicators studied, and access to services even declined in some provinces (Fig. 2). For example, compared with 2008, coverage of SBA decreased in 2012 in the Eastern Cape and North West Provinces. Gauteng had widened socioeconomic inequalities in the indicator ANC attendance before 20 weeks, while the relative inequalities in this variable narrowed in Western Cape, Eastern Cape and KwaZulu Natal (Fig. 2). Disparities also widened in 2012 compared to 2008 for self-reported health status in the North West and Gauteng provinces, but were narrower in the Western Cape. Inequalities for planned pregnancy were also wider in the Free State and North West provinces in 2012 compared to 2008. Lastly, in the two provinces with the highest HIV prevalence, KwaZulu Natal and Mpumalanga, only 21.8 % and 25.2 % of pregnancies were planned respectively.

Overall, the Eastern Cape Province still has the poorest access to services for many measures, aside from HIV testing, which had approximated national levels by 2012. Compared with the national average, in the Eastern Cape, 9.0 % fewer women had four or more ANC visits; 18.0 % less attended ANC before 20 weeks of pregnancy; and SBA coverage was 8.2 % lower. Moreover, in contrast with other provinces, coverage of some services in the Eastern Cape actually declined between 2008 and 2012. Planned pregnancy in the province decreased from 38.1 % in 2008 to 18.8 % in 2012 and HIV prevalence rose from 18.6 to 25.1 %.

## Discussion

The extraordinarily high levels of inequalities noted in 2008 persist and were even exacerbated in many of the services studied. Several underlying disparities in the social determinants of health also worsened. Moreover, substantial declines took place in the overall coverage of ANC services and in planned pregnancy in the country. Encouragingly, however, some notable advances have occurred in access to maternal health services in the country as a whole. HIV testing coverage rose nationally, as did early ANC attendance and the likelihood of having a doctor present at birth. Also, inequalities detected in health status and in HIV testing in 2008 were no longer present by 2012. Improvements in health status may be due to the high coverage of ART, and consequent gains in health among HIV-infected women, who are predominately in QI and QII.

Inequalities experienced by several PROGRESS-Plus groups warrant specific mention. Overall, compared with other race groups, Black African women had poorer access to services and health outcomes on several indicators, most notably early ANC attendance, skilled birth attendance, planned pregnancy and HIV prevalence. These findings are particularly concerning given the strong links between these indicators and the risk for mother-to-child transmission of HIV (MTCT). As in 2008, education level was linked with almost all measures of access to services and health outcomes. Though the proportion of women completing secondary school in lower economic groups appears to be rising, these are matched by worsening inequities in employment. Also noteworthy, rural areas experienced several deficiencies in service delivery, particularly in access to a SBA. Poorer socio-economic groups are increasingly concentrated within the Eastern Cape, and several services there actually deteriorated over the study period. Service delivery deficiencies were also noted in North West Province, albeit to a lessor extent than the Eastern Cape. Both these provinces are plagued with corruption [34, 35].

Differentials in coverage of services must also be interpreted in the light of markedly asymmetric needs between population groups, as measured by HIV status, for example. HIV prevalence in the poorest quartile is 20 percentage points higher than in the wealthiest women, signalling the chronic deficiencies in access to high-quality health, education and social services in the country. HIV remains the preeminent risk factor for maternal morbidity and mortality in the country [36, 37] and infected women require more, not equal, levels of services. It is, however, precisely those with the highest levels of need that remain underserved. Still 5 percent of women have never accessed HIV testing, which should be universally provided for all pregnant women in the country [38]. Non-testing is even higher in some groups with a high HIV prevalence, many of whom might mistakenly perceive themselves to be at low risk, as they are older, married housewives, or feel protected by living within a rural traditional society. Information campaigns could focus on addressing these misperceptions. Gaps in HIV testing among older women also perhaps reflect the difficulties of an often considerably younger peer counsellor in discussing HIV testing and sexual matters with older women.

Timely and frequent attendance at ANC and for facility birth are especially important for preventing MTCT and conditions such as hypertension, anaemia, and fetal alcohol spectrum disorders, which require early detection and repeated interventions during pregnancy. As ART is now widely available [39], the most critical factor which determines whether a woman transmits HIV to her infant is the duration of ART during pregnancy [40, 41]. Demand- and supply-side interventions could reverse the concerning disparities noted and overall gaps in these services.

The persistent, even escalating, socially-determined inequities in service access support the call for state support during pregnancy in South Africa [42]. Moreover, the widening of unemployment differentials indicates that income-related inequities among pregnant women are actually worsening. The existing child support grant in South Africa, widely credited with improving child health in the country [43], should be initiated during pregnancy to alleviate the indirect costs of accessing services, such as transport and childcare while away from the family [42]. Grants given during pregnancy have raised service utilisation in many Latin American and South East Asian countries, and have led to marked improvements in maternal and child health outcomes [44].

Attention paid to family planning has risen in recent years, both globally and within South Africa [45]. Increases in unintended pregnancy, especially among young women and scholars, as well as a widening of inequalities in this outcome, indicate that a focus on family planning is long overdue. Improvements in family planning services would lower maternal, neonatal and infant mortality, and assist in reaching the MTCT elimination targets (nearly 70 % of pregnancies among HIV-infected women were unplanned in both surveys) [46]. It will be important to monitor whether family planning services have improved following the Contraceptive Policy introduced in South Africa in 2013.

### Limitations

Aside from biological measures of HIV status, many outcomes were self-reported, and subject to social desirability and recall biases. Self-assessed health status may be especially vulnerable to misclassification. Although this measure is associated with morbidity and predicts mortality, its validity may vary across population groups [47–49]. As a further limitation, we did not adjust the socio-economic inequality measures for potential confounding variables, such as race, education and geographical areas. Indeed, the analysis largely focused on impacts of socio-economic inequalities, and only presents salient findings related to the other PROGRESS-Plus groups.

The proportion ineligible or declining participation in the surveys was substantial. Plausibly, those declining enrolment may, for example, have had different health-seeking behaviours than those who participated. Moreover, wealthier and white race groups were more likely to decline survey participation [10], groups that might incur specific forms of measurement bias. For example, wealthy women who visited a private-sector obstetrician during pregnancy may have reported not having received antenatal care during pregnancy. Finally, in future studies, measuring distance to a facility could provide a better assessment of geographical access than simple rural and urban dichotomies. This information and data from tools such as Global Positioning Systems could be used for pinpointing poorly performing areas.

## Conclusions

The most striking finding is the widening of both absolute and relative inequalities in several measures of access to care. Even in measures where inequalities did not rise, substantial disparities persist. Encouragingly, differences in overall health status between quartiles narrowed.

This analysis provides actionable information by identifying which interventions and groups require most attention. Considerable gains could be made by targeting underserved groups, as well as the factors underlying the differences in access, such as poor quality services and unsatisfactory patient experiences. Clearly, the continued deficiencies, even deterioration in service delivery in the Eastern Cape and North West Provinces warrant attention. Furthermore, to reach the country’s targets for elimination of HIV infection in children, government should focus on improving access to HIV testing in groups which have relatively low HIV testing coverage, but high HIV prevalence, such as older women. Strengthening of family planning services, especially for teenagers, scholars and other low uptake groups would also reduce HIV infections in children, among other sizable benefits.

Though the health system bears responsibility for narrowing gaps in access, factors outside the health sector, such as unemployment, also account for demand for care and population health more broadly [2, 50]. Demand side interventions could incentivise early and frequent ANC visits. This might be achieved by beginning the child support grant in pregnancy and requiring an ANC card as part of grant enrolment procedures. Finally, policy makers will need to choose between prioritising further overall improvements in health services (noted on several indicators in this study), or redressing the uneven improvements that have taken place.

## Additional files

Additional file 1: Table S1. Results on inequalities in access to maternal health services across different populations groups in South Africa. (DOCX 35 kb) Additional file 2: Table S2. Results on inequalities in maternal health outcomes across different populations groups in South Africa. (DOCX 26 kb)
