# Supplementary material for: Growing inequities in maternal health in South Africa: a comparison of serial national household surveys
Source: BMC Pregnancy Childbirth. 2016 Sep 1;16(1):256. doi: 10.1186/s12884-016-1048-z (PMC5007803; doi:10.1186/s12884-016-1048-z)
Supplement: Additional file 1: Table S1. — Results on inequalities in access to maternal health services across different populations groups in South Africa. (DOCX 35 kb) [file 12884_2016_1048_MOESM1_ESM.docx]

**Table S1 Inequities in access to maternal health services across different populations groups in South Africa**

| **Variable  (N; P-value)** | **Received any ANC** | | **Attended ≥4 ANC visits** | | **ANC visits <20 weeks gestation** | | **Skilled birth attendant** | | **Doctor at childbirth** | | **Offered HIV testing** | | **Had HIV test in past 2 years** | | **Never had HIV test** | |
| --- | --- | --- | --- | --- | --- | --- | --- | --- | --- | --- | --- | --- | --- | --- | --- | --- |
|  | 2008 | 2012 | 2008 | 2012 | 2008 | 2012 | 2008 | 2012 | 2008 | 2012 | 2008 | 2012 | 2008 | 2012 | 2008 | 2012 |
| **Total** | 97.0 | 90.2 | 85.6 | 87.2 | 45.1 | 67.2 | 95.3 | 95.5 | 27.8 | 34.4 | 92.9 | 97.8 | 87.0 | 97.2 | 8.5 | 4.0 |
| **Socio-economic quartile** | ***1248*** | **1469** | ***1251*** | **1220 *0.003*** | ***1250 0.017*** | **1309 *<0.001*** | ***1263 <0.001*** | **1455 <0.001** | ***1263 <0.001*** | **1455 <0.001** | ***1245 0.081*** | **1307 *0.002*** | ***1112*** | **1543** | ***1112*** | **1607** |
| QI | 98.5 | 89.6 | 84.8 | 80.5 | 36.2 | 56.0 | 90.9 | 90.5 | 17.5 | 17.3 | 94.2 | 98.9 | 89.9 | 96.9 | 8.5 | 3.8 |
| QII | 97.2 | 87.7 | 86.4 | 85.2 | 43.4 | 70.0 | 94.9 | 97.0 | 20.5 | 29.7 | 92.6 | 98.7 | 85.2 | 98.6 | 10.2 | 2.9 |
| QIII | 95.9 | 92.5 | 83.6 | 91.8 | 51.4 | 67.0 | 99.1 | 98.0 | 35.2 | 40.7 | 94.7 | 98.7 | 86.0 | 95.7 | 7.1 | 2.8 |
| QIV | 94.2 | 92.2 | 90.5 | 96.5 | 59.7 | 86.1 | 99.4 | 98.9 | 61.5 | 71.0 | 85.8 | 91.9 | 87.1 | 98.2 | 7.1 | 8.8 |
| **Age (years)** | ***1256*** | ***1496 0.028*** | ***1260*** | ***1240 0.034*** | ***1258*** | ***1331 0.019*** | ***1269*** | ***1482 0.052*** | ***1269*** | ***1482*** | ***1253 0.006*** | ***1329*** | ***1113 0.008*** | ***1570 0.029*** | ***1113 0.032*** | ***1635*** |
| 15-19 | 100.0 | 92.6 | 82.9 | 79.3 | 45.9 | 52.6 | 95.1 | 98.6 | 23.2 | 30.4 | 96.1 | 97.5 | 87.5 | 97.6 | 12.2 | 5.4 |
| 20-29 | 96.7 | 88.5 | 87.2 | 85.7 | 42.7 | 66.6 | 94.8 | 95.9 | 25.8 | 32.8 | 94.7 | 98.7 | 90.8 | 98.3 | 6.2 | 2.3 |
| 30-39 | 95.6 | 93.9 | 84.6 | 92.3 | 46.8 | 73.5 | 97.3 | 94.8 | 32.8 | 38.3 | 89.5 | 96.5 | 83.7 | 95.6 | 8.9 | 5.6 |
| 40-55 | 100.0 | 82.9 | 85.2 | 90.1 | 59.3 | 64.1 | 93.4 | 87.5 | 27.8 | 32.7 | 82.5 | 98.3 | 68.9 | 91.7 | 24.3 | 8.0 |
| **Place of residence** | ***1261 0.003*** | ***1496*** | ***1264*** | ***1240*** | ***1263 0.035*** | ***1331 0.042*** | ***1277 <0.001*** | ***1482 0.002*** | ***1277 <0.001*** | ***1482 <0.001*** | ***1258*** | ***1329*** | ***1117*** | ***1570*** | ***1117*** | ***1635*** |
| Urban formal | 95.9 | 91.0 | 86.4 | 89.6 | 50.4 | 70.6 | 99.2 | 97.9 | 36.9 | 48.9 | 92.4 | 97.3 | 86.5 | 96.4 | 8.5 | 3.3 |
| Urban informal | 97.0 | 90.8 | 89.2 | 86.2 | 48.0 | 61.4 | 95.4 | 98.3 | 31.0 | 24.9 | 94.0 | 99.5 | 88.5 | 98.5 | 5.6 | 4.1 |
| Rural formal | 89.8 | 86.7 | 79.9 | 87.0 | 47.9 | 82.8 | 85.8 | 94.3 | 25.1 | 48.8 | 84.2 | 91.7 | 81.6 | 94.8 | 8.7 | 13.7 |
| Rural informal | 99.5 | 89.8 | 85.2 | 85.2 | 38.2 | 63.4 | 92.9 | 92.4 | 17.0 | 20.0 | 94.8 | 98.7 | 88.6 | 97.8 | 9.4 | 3.6 |
| **Province** | ***1261*** | ***1496 0.055*** | ***1264*** | ***1240*** | ***1263*** | ***1331 0.003*** | ***1277 0.007*** | ***1482 0.014*** | ***1277 0.001*** | ***1482 <0.001*** | ***1258*** | ***1329*** | ***1117*** | ***1570*** | ***1117*** | ***1635*** |
| Eastern Cape | 98.3 | 91.1 | 79.5 | 78.3 | 30.8 | 49.2 | 96.3 | 87.2 | 28.4 | 20.4 | 96.9 | 99.0 | 88.3 | 95.4 | 9.1 | 3.6 |
| Free State | 98.4 | 95.8 | 92.9 | 85.7 | 49.1 | 74.0 | 86.4 | 96.8 | 19.3 | 37.3 | 93.9 | 100.0 | 89.2 | 98.3 | 4.7 | 4.7 |
| KwaZulu Natal | 97.1 | 89.5 | 88.7 | 90.2 | 42.6 | 71.4 | 94.9 | 95.3 | 29.5 | 38.5 | 94.7 | 99.1 | 88.8 | 98.7 | 7.8 | 1.3 |
| Gauteng | 94.5 | 90.7 | 83.5 | 86.2 | 44.1 | 66.7 | 98.6 | 99.2 | 36.8 | 46.1 | 91.2 | 95.5 | 85.6 | 96.9 | 8.1 | 5.7 |
| Limpopo | 100.0 | 82.8 | 87.3 | 86.6 | 55.1 | 66.5 | 90.8 | 95.0 | 11.0 | 19.2 | 88.7 | 97.3 | 86.8 | 99.3 | 10.4 | 5.1 |
| Mpumalanga | 98.2 | 87.0 | 89.1 | 95.4 | 48.5 | 76.3 | 93.9 | 96.1 | 18.0 | 23.3 | 91.9 | 99.5 | 82.1 | 97.4 | 15.5 | 3.2 |
| North West | 95.3 | 99.2 | 84.0 | 88.9 | 52.8 | 61.7 | 96.0 | 95.5 | 25.5 | 18.3 | 90.2 | 100.0 | 85.5 | 97.4 | 8.6 | 4.7 |
| Northern Cape | 99.4 | 95.8 | 93.5 | 94.4 | 68.2 | 71.0 | 99.4 | 97.1 | 21.7 | 38.9 | 98.4 | 99.0 | 94.4 | 97.5 | 4.1 | 0.9 |
| Western Cape | 94.8 | 90.7 | 83.7 | 90.0 | 47.1 | 78.1 | 100.0 | 95.8 | 42.3 | 51.6 | 92.2 | 96.2 | 88.6 | 92.4 | 3.5 | 3.4 |
| **Race** | ***1254 <0.001*** | ***1492*** | ***1257*** | ***1237*** | ***1256*** | ***1328 <0.001*** | ***1270*** | ***1478; .025*** | ***1270 0.001*** | ***1478 <0.001*** | ***1251 <0.001*** | ***1326; <0.001*** | ***1112*** | ***1566*** | ***1112 0.026*** | ***1631 <0.001*** |
| Black African | 98.3 | 90.0 | 85.9 | 86.6 | 43.1 | 64.2 | 94.8 | 94.9 | 23.5 | 28.4 | 94.8 | 98.9 | 87.5 | 97.4 | 9.1 | 2.9 |
| White | 74.8 | 91.5 | 72.6 | 90.8 | 60.3 | 97.9 | 100.0 | 96.4 | 67.8 | 89.7 | 62.4 | 75.4 | 75.8 | 94.8 | 2.0 | 30.2 |
| Coloured | 97.4 | 93.1 | 91.4 | 90.2 | 53.5 | 78.0 | 96.7 | 99.2 | 37.6 | 57.6 | 95.1 | 97.7 | 90.4 | 94.1 | 3.2 | 2.4 |
| Indian/Asian | 89.8 | 88.3 | 82.0 | 97.6 | 65.1 | 89.3 | 100.0 | 99.7 | 75.5 | 90.8 | 70.8 | 97.6 | 83.1 | 98.3 | 8.7 | 4.6 |
| **Highest education** | ***740 0.070*** | ***1345*** | ***741*** | ***1122*** | ***744*** | ***1197 0.026*** | ***751 <0.001*** | ***1335 <0.001*** | ***7510*** | ***1335 <0.001*** | ***739*** | ***1197 0.017*** | ***1113*** | ***1404*** | ***1113 0.016*** | ***1463*** |
| Grade 0-3 | 92.5 | 86.2 | 80.8 | 72.9 | 33.6 | 71.1 | 86.3 | 73.2 | 30.4 | 13.0 | 87.5 | 98.9 | 76.4 | 96.5 | 19.9 | 4.1 |
| Grade 4-7 | 97.6 | 91.9 | 84.5 | 80.6 | 42.2 | 63.9 | 93.8 | 88.5 | 9.1 | 17.8 | 96.5 | 98.5 | 93.1 | 93.7 | 3.9 | 7.7 |
| Grade 8-11 | 98.6 | 88.1 | 84.4 | 83.7 | 40.6 | 59.7 | 94.6 | 95.9 | 17.6 | 28.6 | 94.7 | 98.5 | 85.0 | 97.3 | 10.4 | 3.3 |
| Grade 12 | 96.6 | 91.6 | 86.0 | 90.1 | 52.1 | 73.7 | 99.9 | 98.8 | 44.6 | 41.4 | 93.4 | 98.4 | 88.3 | 97.4 | 7.9 | 2.4 |
| Tertiary | 86.8 | 91.8 | 85.6 | 94.7 | 35.3 | 78.9 | 99.7 | 99.4 | 70.6 | 63.1 | 87.6 | 90.7 | 90.7 | 97.7 | 1.5 | 8.8 |
| **Employment** | ***721 0.001*** | ***1467*** | ***722 0.004*** | ***1215 0.045*** | ***725 0.026*** | ***1305 0.009*** | ***732*** | ***1452*** | ***732 <0.001*** | ***1452 <0.001*** | ***720*** | ***1302*** | ***1098*** | ***1541*** | ***1098*** | ***1604*** |
| Housewife | 96.5 | 91.4 | 87.3 | 87.4 | 48.3 | 71.4 | 91.8 | 91.7 | 21.8 | 41.9 | 91.7 | 98.4 | 86.9 | 97.0 | 8.3 | 7.1 |
| Unemployed not seeking work | 99.2 | 85.9 | 75.0 | 81.9 | 31.2 | 53.3 | 98.6 | 92.3 | 15.4 | 26.5 | 96.3 | 97.5 | 91.9 | 98.2 | 8.1 | 5.9 |
| Unemployed-seeking work | 98.7 | 88.2 | 90.3 | 84.3 | 41.5 | 68.1 | 95.4 | 96.3 | 27.1 | 22.5 | 95.8 | 98.6 | 86.6 | 98.3 | 8.9 | 2.2 |
| Informal sector, self-employed | 99.1 | 88.2 | 91.7 | 97.1 | 71.5 | 81.4 | 94.8 | 92.9 | 37.5 | 65.0 | 95.6 | 94.0 | 87.2 | 92.3 | 2.6 | 2.4 |
| Student Learner | 100.0 | 89.2 | 67.5 | 81.7 | 39.5 | 49.1 | 98.2 | 94.7 | 18.3 | 26.5 | 94.9 | 98.9 | 89.2 | 98.8 | 8.6 | 1.6 |
| Part employed | 99.5 | 96.8 | 94.0 | 97.2 | 33.3 | 73.9 | 99.3 | 100.0 | 19.9 | 49.6 | 95.5 | 100.0 | 90.5 | 99.6 | 7.0 | 4.2 |
| Full employed | 87.0 | 95.8 | 75.0 | 92.5 | 50.7 | 74.5 | 98.9 | 98.9 | 65.0 | 52.8 | 86.4 | 94.9 | 85.2 | 94.8 | 9.7 | 6.7 |
| Other | 100.0 | 82.4 | 100.0 | 91.2 | 0.0 | 50.8 | 100.0 | 89.4 | 31.0 | 36.4 | 100.0 | 98.6 | 78.7 | 93.2 | 11.5 | 4.9 |
| **Marital Status** | ***730*** | ***1480*** | ***731*** | ***1227*** | ***734*** | ***1317 0.023*** | ***741*** | ***146 ;0.015*** | ***741*** | ***1466 <0.001*** | ***729*** | ***1315*** | ***1115*** | ***1553*** | ***1115*** | ***1618 0.004*** |
| Single | 98.3 | 90.9 | 84.2 | 87.1 | 40.6 | 63.6 | 95.4 | 96.6 | 24.6 | 27.0 | 95.2 | 99.0 | 88.0 | 97.4 | 9.5 | 2.0 |
| Married/cohabiting | 95.3 | 89.5 | 87.1 | 87.3 | 47.9 | 72.5 | 96.3 | 94.4 | 34.7 | 43.2 | 91.9 | 96.3 | 86.4 | 96.8 | 6.6 | 6.4 |
| Divorced/widow | 100.0 | 93.0 | 84.2 | 98.4 | 60.3 | 65.9 | 91.0 | 83.6 | 34.4 | 26.7 | 93.9 | 100.0 | 78.5 | 92.5 | 11.4 | 3.4 |
| **HIV infection** | ***850*** | ***1226*** | ***852*** | ***1008*** | ***851*** | ***1082 0.013*** | ***860 0.003*** | ***1213*** | ***860 0.048*** | ***1213 0.011*** | ***848*** | ***1084*** | ***898*** | ***1294 0.052*** | ***898*** | ***1340*** |
| No HIV infection | 97.2 | 89.2 | 84.8 | 84.5 | 45.7 | 63.5 | 96.3 | 94.7 | 27.2 | 34.8 | 93.6 | 98.5 | 88.0 | 98.0 | 7.8 | 2.8 |
| HIV infection | 99.1 | 91.8 | 90.6 | 91.1 | 46.2 | 75.2 | 88.0 | 96.1 | 17.0 | 23.5 | 91.3 | 98.5 | 85.1 | 94.9 | 11.0 | 3.9 |

*P* value assesses the distribution of access within a population group in each survey. Only *P* values <0.05 are shown
