# Supplementary material for: Growing inequities in maternal health in South Africa: a comparison of serial national household surveys
Source: BMC Pregnancy Childbirth. 2016 Sep 1;16(1):256. doi: 10.1186/s12884-016-1048-z (PMC5007803; doi:10.1186/s12884-016-1048-z)
Supplement: Additional file 2: Table S2. — Results on inequalities in maternal health outcomes across different populations groups in South Africa. (DOCX 26 kb) [file 12884_2016_1048_MOESM2_ESM.docx]

**Table S2 Inequities in maternal health outcomes across different populations groups in South Africa**

| **Variable (N; P-value)** | **Planned pregnancy** | | **≥5 Children** | | **Poor-fair health status** | | **HIV infection** | |
| --- | --- | --- | --- | --- | --- | --- | --- | --- |
|  | 2008 | 2012 | 2008 | 2012 | 2008 | 2012 | 2008 | 2012 |
| **Total** | 44.6 | 34.7 | 5.2 | 6.4 | 12.6 | 13.2 | 22.8 | 24.5 |
| **Socio-economic quartile** | ***1105 0.029*** | **1459 *<0.001*** | ***1013 0.017*** | **1544 *<0.001*** | ***1113 0.014*** | **1608** | **896 0.002** | **1333 0.002** |
| QI | 43.2 | 22.4 | 8.6 | 13.5 | 15.5 | 11.8 | 26.8 | 30.7 |
| QII | 40.7 | 31.0 | 5.2 | 5.0 | 16.2 | 13.0 | 30.0 | 26.1 |
| QIII | 43.8 | 42.8 | 3.0 | 2.4 | 8.0 | 16.2 | 13.9 | 22.2 |
| QIV | 65.7 | 53.0 | 0.4 | 2.0 | 5.0 | 10.8 | 6.2 | 9.0 |
| **Age (years)** | ***1108 <0.001*** | ***1485 <0.001*** | ***1015 <0.001*** | ***1570 <0.001*** | ***1114 0.001*** | ***1636 0.018*** | **897 0.032** | **1348 <0.001** |
| 15-19 | 12.2 | 8.7 | 0.0 | 0.0 | 7.9 | 5.5 | 11.6 | 8.4 |
| 20-29 | 42.2 | 32.2 | 1.4 | 0.9 | 9.1 | 11.9 | 22.6 | 22.7 |
| 30-39 | 56.9 | 46.4 | 9.2 | 11.4 | 18.6 | 17.0 | 27.9 | 35.1 |
| 40-55 | 52.9 | 40.9 | 26.5 | 48.3 | 18.9 | 19.1 | 14.5 | 21.3 |
| **Place of residence** | ***1111 0.014*** | ***1485 <0.001*** | ***1019 0.031*** | ***1570 0.036*** | ***1118 0.031*** | ***1636*** | **900 *0.006*** | ***1348 0.001*** |
| Urban formal | 48.6 | 46.0 | 2.5 | 3.5 | 8.5 | 14.2 | 17.4 | 17.4 |
| Urban informal | 40.5 | 29.8 | 8.7 | 6.1 | 14.3 | 16.6 | 36.2 | 35.7 |
| Rural formal | 58.0 | 37.1 | 5.5 | 9.1 | 14.7 | 10.9 | 16.2 | 21.7 |
| Rural informal | 37.2 | 23.5 | 6.7 | 9.0 | 16.6 | 11.4 | 25.2 | 29.3 |
| **Province** | ***1111 <0.001*** | ***1485 <0.001*** | ***1019*** | ***1570 0.027*** | ***1118 0.038*** | ***1636*** | **900 *0.003*** | ***1348 0.045*** |
| Eastern Cape | 38.1 | 18.8 | 4.3 | 11.8 | 13.2 | 9.7 | 18.6 | 25.1 |
| Free State | 51.7 | 48.5 | 1.9 | 7.5 | 7.7 | 17.7 | 10.2 | 31.3 |
| Gauteng | 56.5 | 51.8 | 5.5 | 3.9 | 9.0 | 15.8 | 23.1 | 19.8 |
| KwaZulu Natal | 25.3 | 21.8 | 7.0 | 3.0 | 20.2 | 8.8 | 35.5 | 31.4 |
| Limpopo | 52.0 | 37.5 | 6.9 | 6.3 | 6.9 | 11.1 | 18.5 | 22.0 |
| Mpumalanga | 45.5 | 25.2 | 7.4 | 13.8 | 18.1 | 17.1 | 34.5 | 35.7 |
| North West | 53.2 | 32.2 | 1.5 | 9.0 | 10.5 | 13.1 | 17.6 | 24.4 |
| Northern Cape | 48.7 | 37.8 | 8.1 | 3.1 | 12.3 | 7.6 | 11.6 | 18.6 |
| Western Cape | 43.7 | 32.1 | 0.0 | 3.1 | 10.7 | 17.4 | 10.1 | 10.0 |
| **Race** | ***1106 <0.001*** | ***1481 0.007*** | ***1015*** | ***1566*** | ***1113*** | ***1632*** | **898 *<0.001*** | ***1345 <0.001*** |
| Black African | 42.9 | 32.9 | 5.7 | 6.7 | 13.2 | 13.3 | 25.7 | 27.1 |
| White | 80.9 | 61.1 | 0.0 | 6.0 | 7.4 | 9.2 | 0.0 | 6.9 |
| Coloured | 38.6 | 35.9 | 1.9 | 3.9 | 7.8 | 16.6 | 3.4 | 7.2 |
| Indian/Asian | 57.9 | 39.5 | 0.0 | 1.2 | 1.0 | 2.6 | 0.0 | 4.1 |
| **Highest education** | ***1107 0.010*** | ***1338 0.001*** | ***1016 <0.001*** | ***1411 <0.001*** | ***1114 <0.001*** | ***1465 0.023*** | **898 *0.006*** | ***1219 0.004*** |
| Grade 0-3 | 32.8 | 38.6 | 26.9 | 28.7 | 37.6 | 17.4 | 39.5 | 37.9 |
| Grade 4-7 | 43.1 | 22.3 | 14.2 | 22.7 | 17.2 | 22.6 | 20.1 | 29.6 |
| Grade 8-11 | 38.2 | 26.7 | 5.1 | 5.0 | 12.7 | 11.3 | 28.7 | 27.1 |
| Grade 12 | 49.3 | 37.4 | 0.3 | 1.3 | 11.0 | 14.2 | 14.0 | 21.1 |
| Tertiary | 61.7 | 53.7 | 0.0 | 1.6 | 2.7 | 6.3 | 13.3 | 3.9 |
| **Employment** | ***1092 <0.001*** | ***1455 <0.001*** | ***1004 <0.001*** | ***1540 0.011*** | ***1099 <0.001*** | ***1605*** | **886 0.005** | ***1324 0.001*** |
| Housewife | 62.0 | 54.6 | 10 | 14.4 | 12.2 | 12.1 | 24.6 | 27.2 |
| Unemployed not seeking work | 31.8 | 22.5 | 1.5 | 6.8 | 8.6 | 10.7 | 16.5 | 19.3 |
| Unemployed-seeking work | 33.8 | 25.1 | 6.9 | 5.5 | 12.5 | 13.5 | 30.3 | 31.1 |
| Informal sector, self-employed | 58.8 | 52.3 | 5.1 | 6.6 | 15.5 | 7.6 | 31.3 | 30.4 |
| Student Learner | 14.6 | 13.9 | 0.0 | 1.1 | 5.9 | 8.4 | 40.5 | 9.0 |
| Part employed | 50.4 | 34.5 | 1.0 | 6.5 | 18.7 | 18.2 | 10.9 | 26.8 |
| Full employed | 62.9 | 59.1 | 0.3 | 3.1 | 9.9 | 15.7 | 9.7 | 12.1 |
| Other | 14.4 | 12.9 | 6.4 | 11.2 | 70.7 | 21.5 | 10.2 | 25.3 |
| **Marital Status** | ***1109 <0.001*** | ***1469 <0.001*** | ***1017 <0.001*** | ***1554 <0.001*** | ***1116*** | ***1619*** | **898 *<0.001*** | ***1334*** |
| Single | 30.4 | 22.3 | 1.8 | 3.6 | 14.4 | 13.1 | 28.7 | 26.2 |
| Married/cohabiting | 65.6 | 51.8 | 9.8 | 8.8 | 9.7 | 12.4 | 13.4 | 23.5 |
| Divorced/widow | 38.6 | 16.8 | 5.6 | 21.2 | 19 | 15.5 | 25.3 | 10.2 |
| **HIV infection** | ***891*** | ***1217*** | ***817*** | ***1284*** | ***899 <0.001*** | ***1337*** |  |  |
| No HIV infection | 42.4 | 30.3 | 5.8 | 6.4 | 9.0 | 12.3 |  |  |
| HIV infection | 31.7 | 32.2 | 4.1 | 10.2 | 25.1 | 20.2 |  |  |
